# Impact of seed priming with Selenium nanoparticles on germination and seedlings growth of tomato

**DOI:** 10.1038/s41598-024-57049-3

**Published:** 2024-03-20

**Authors:** Ezequiel García-Locascio, Edgardo I. Valenzuela, Pabel Cervantes-Avilés

**Affiliations:** Escuela de Ingeniería y Ciencias, Tecnologico de Monterrey, Reserva Territorial Atlixcáyotl, CP 72453 Puebla, Pue México

**Keywords:** Nano-agrochemicals, *Solanum lycopersicum*, Chlorophyll, Total antioxidant capacity, Proline, Biotechnology, Plant sciences, Environmental sciences

## Abstract

Poor germination and seedlings growth can lead to significant economic losses for farmers, therefore, sustainable agricultural strategies to improve germination and early growth of crops are urgently needed. The objective of this work was to evaluate selenium nanoparticles (Se NPs) as nanopriming agents for tomato (*Solanum lycopersicum*) seeds germinated without stress conditions in both trays and Petri dishes. Germination quality, seedlings growth, synergism-antagonism of Se with other elements, and fate of Se NPs, were determined as function of different Se NPs concentrations (1, 10 and 50 ppm). Results indicated that the germination rate in Petri dishes improved with 10 ppm, while germination trays presented the best results at 1 ppm, increasing by 10 and 32.5%, respectively. Therefore, seedlings growth was measured only in germination trays. Proline content decreased up to 22.19% with 10 ppm, while for same treatment, the total antioxidant capacity (TAC) and total chlorophyll content increased up to 38.97% and 21.28%, respectively. Antagonisms between Se with Mg, K, Mn, Zn, Fe, Cu and Mo in the seed were confirmed. In the case of seedlings, the N content decreased as the Se content increased. Transmission Electron Microscopy (TEM) imaging confirmed that Se NPs surrounded the plastids of the seed cells. By this finding, it can be inferred that Se NPs can reach the embryo, which is supported by the antagonism of Se with important nutrients involved in embryogenesis, such as K, Mg and Fe, and resulted in a better germination quality. Moreover, the positive effect of Se NPs on total chlorophyll and TAC, and the negative correlation with proline content with Se content in the seed, can be explained by Se NPs interactions with proplastids and other organelles within the cells, resulting with the highest length and fresh weight when seeds were exposed to 1 ppm.

## Introduction

Achieving global food security and preventing further environmental degradation requires the implementation of sustainable agricultural practices^1^. In fact, agriculture is essential for the three pillars of sustainability: environment, society, and economy^2^. Tomato is a pivotal crop in agriculture, it is the second most important vegetable crop and the most cultivated in the S*olanaceae* family^3–5^. Therefore, researchers have focused on developing sustainable agricultural practices, including the addition of nanoagrochemicals for tomato and other important crops^6–8^. In agriculture, nanoagrochemicals can offer advantages compared to conventional agrochemicals, such as reducing the negative impact to the environment, increasing plant resilience to biotic and abiotic stress, minimizing pollution, and using less water^6^. These benefits are also related to the delivery method, with foliar and soil applications as the most common methods to deliver nanoagrochemicals^9,10^. Seed priming is an innovative method to deliver the nanoagrochemicals that can be used to enhance the imbibition capacity and metabolic system of the seeds to increase germination, stress resilience, or pest resistance^11,12^. Recent studies about seed priming have focused in improving the stress resilience of different crops, for instance, it was recently reported that nanopriming seeds of garden pea (*Pisum sativum*) with iron oxide NPs improved the growth, biochemical and yield parameters under drought stress conditions^13^. Furthermore, similar positive results were reported in seed priming experiments of canola (*Brassica napus*) exposed to CaO nanoparticles (NPs), which increased the germination percentage and the seedling fresh weight^14^.

Nanoagrochemicals in tomato have been applied as nanopesticides and nanofertilizers. Nanofertilizers include seed primers and aim to increase germination and growth parameters to improve tomato plants resilience to abiotic stress, such as drought, heat, and salt, among others^15^. While nanopesticides have also been tested against the most important phytopathogens for this crop, such as *Fusarium oxysporum f. sp. lycopersici*, X*anthomonas perforans*, *Pseudomonas syringae, Botrytis cinerea, Phytophthora infestans, and Clavibacter michiganensis*, with excellent antibacterial and antifungal activity compared to their bulk counterparts^16,17^. Amongst the NPs used for tomato crops, many different chemical compositions have been tested as nanofertilizers and nanopesticides for tomato crops with contradictory results, such as Mg, Fe, Ce, Ti, Si, S, C, and Se, including the most common NPs such as Cu-based, and Zn-based NPs^18–22^. These last compositions have been more related to positive results as priming agents in tomato seeds, increasing the germination rate and overall germination quality^23–25^. In general, some of the reported benefits of NPs in tomato include, but are not limited to, a higher germination rate, increases in biomass amount, chlorophyll content, and yield. Negative effects have also been reported, such as inhibition of germination, lower chlorophyll contents, oxidative stress, amongst others. However, the effects of NPs based on other chemical composition might differ due to concentration and physicochemical properties of the NPs, the delivery method used, and exposure conditions.

Se NPs have been found to be beneficial nanofertilizer for different crops such as (optimal concentration in parentheses): rapeseed (*Brassica napus*) (28.34 ppm)^26^, cucumber (*Cucumis sativus*) (25 ppm)^27^, coffee (*Coffea arabica*) (120 ppm)^28^, pomegranate (*Punica granatum*) (0.378 ppm)^29^, amongst others. In tomato, Se NPs have been used to alleviate stress symptoms and diseases, e.g., it was recently reported that zero-valent Se (Se^0^) NPs applied to the soil-root interface at 5 – 10 ppm resulted in slight increases of growth parameters in tomato heat-stressed plants, although treatments with 1 and 25 ppm lacked efficiency^30^. Bio-synthesized Se NPs also helped to increase resistance and defense responses of tomato plants against late blight disease (*Phytophthora infestans*) through seed priming with 100 ppm, achieving up to 72.9% protection against the phytopathogen and other positive secondary effects, such as increasing the germination by 22%^31^. Tomato seeds primed with a nanocomposite of mesoporous Se NPs also increased their germination up to 11% at 100 ppm, while the nanocomposite exhibited great antifungal efficiency against tomato gray mold (*Botrytis cinerea*) disease^32^. Although positive effects of Se NPs in tomato have been reported, studies have focused on stress alleviation, disease prevention and treatment when plants are in vegetative stage, while studies germinating tomato seeds without abiotic or biotic stress are very scarce and needed. The application of Se NPs as a nanopriming agent in healthy tomato seeds without induced stress will help to understand their effects under natural conditions. The main objective of this study was to investigate the effects of nanopriming tomato seeds with Se NPs as function of the germination quality, seedlings growth, synergism-antagonism of Se with other elements, and find the optimal concentration of Se NPs for tomato seed priming. The fate of Se NPs in nanoprimed seeds was also determined by transmission electron microscopy (TEM) imaging.

## Materials and methods

### Se NPs source and characterization

Elemental Se NPs were acquired from ID Nano (México) with a primary particle size of < 50 nm. The morphology of the NPs was determined by transmission electron microscopy (TEM, JEOL JEM 1010). The hydrodynamic diameter and the ζ potential were measured by dynamic light scattering (DLS, Zetasizer Lab, Malvern Panalytical). The localized surface plasmon resonance (LSPR) was determined by UV–vis spectrophotometer (Jenway, 6715 UV)^10,33^. Se NPs stock suspension concentration was 4000 ppm, which was diluted with milliQ water to 1, 10 and 50 ppm, which were experimental concentrations due to values from 1 to 100 ppm presented positive effects in tomato and other crops when applied via different delivery systems as mentioned in the introduction. Stock and experimental suspensions were dispersed by ultrasonication during 30 min at 185W (Branson 5510). After ultrasonication, Se NPs were then sterilized by ultraviolet radiation for 60 min (Labconco Purifier, Class II, type A2, BSC)^34^.

### Tomato seed priming experiments

Commercial tomato Floradade var. seeds were procured from United States of America. The seeds were sterilized with sodium hypochlorite solution at 8% for 10 min, and then immediately washed thrice with MilliQ water. Then, the seeds were added to their corresponding treatment of 1, 10 and 50 ppm of elemental Se NPs (1000 mL of solution per treatment) and vigorously shaken at 250 rpm for 180 min at room temperature (IKA KS 4000 ic control). The primed seeds were germinated using two methods, Petri dishes and germination trays. The seeds were introduced to Petri dishes with distilled water or to germination trays with vermiculite and perlite (2:1 w/w) as substrate. Each experimental treatment was performed in quadruplicate, using 40 seeds for each treatment. MilliQ water was established as the control media for seed priming. The seeds were maintained in dark conditions for 7 days and the germination parameters were measured on the 8th day^13,35^. The fate of Se NPs within the seed is determined through transmission electron microscopy (TEM), while the content of the inorganic elements in the seeds was measured by inductively coupled plasma mass spectrometry (ICP-MS). For these analyses, seeds were digested in 10 mL of HNO_3_ at 70% using a microwave oven system (Mars 6, CEM corp.), where temperature was gradually increased to 200 °C, maintained for 10 min, and then let gradually cool down to room temperature.

### Measurement of seed germination

The parameters measured include germination rate (GR, Eq. (1)), germination potential (GP, Eq. (2)), mean germination time (MGT, Eq. (3)), germination index (GI, Eq. (4)), and vigor index (VI, Eq. (5))^34,35^.

1 $$GR=\left(\frac{ number\, of \,germinated\, seeds}{total\, seeds \,in\, the\, experiment}\right)*100 \%$$

2 $$GP=\left(\frac{ number\, of \,germinated\, seeds\, in\, 5\, days}{total\, seeds\, in\, the \,experiment}\right)*100 \%$$

3 $$MGT=\left(\frac{\begin{array}{c}\Sigma\, number\, of\, germinated\, seeds\, in\, 7 \,days \,*\\ numbers\, of \,days\, since\, the\, start \,of \,the \,experiment\end{array}}{total\, seeds\, in\, the\, experiment}\right)$$

4 $$GI=\left(\frac{\Sigma\, number\, of\, seeds\, germinated\, in\, 8\, days}{number\, of \,days\, of \,germination}\right)$$

5 $$VI=\left(\frac{germination\, index}{average\, of \,tomato \,seedlings \,lenght}\right)$$

### Fate of Se NPs in tomato primed seeds

Standard TEM procedures were conducted to find if Se NPs penetrated the seed coat. Tomato seeds samples were thoroughly washed with MilliQ water and prefixed in 3% glutaraldehyde for 1 h at room temperature. Then, samples were washed in sodium cacodylate buffer, and postfixed in 1% osmium tetroxide at 4 °C for 24 h. The samples were washed with sodium cacodylate buffer and dehydrated in a graded series of ethanol (50–100%). Finally, samples were embedded in epoxy resin (EPON 812), sectioned with an ultramicrotome to 60–70 nm of thickness, and placed in Cu-based grids for TEM observation (TEM, JEOL JEM 1010)^36,37^.

### Tomato seedlings growth

Plant length and fresh weight values were measured 21 days after sowing (21 DAS) for tomato seedlings germinated in trays. Length values were calculated with a thread and a scale (0 – 30 cm) and expressed in centimeters. Fresh weight values were determined with an analytical balance (Ohaus Adventurer) and expressed in milligrams^38^.

#### Total antioxidant capacity (TAC)

TAC was measured to aerial tissue following the Trolox equivalent standard method^39^. Briefly, we reconstituted a Trolox standard solution with dimethyl sulfoxide (DMSO) to prepare a calibration curve. Then, the prepared standard was introduced into a 96 well plate and 100 µL of prepared Cu^2+^ solution was added. Furthermore, samples from whole aerial tissue of the seedlings were diluted with protein mask, and 100 µL of prepared Cu^2+^ solution was added. All samples, standards, and blank (same reagents without plant tissue) were incubated in darkness for 90 min at room temperature. Finally, absorbance was measured at 570 nm and Trolox equivalents were calculated^40^ with Eq. (6), where C represents the Trolox equivalents in nmol/µL, S_a_ is the Trolox equivalent of the sample from standard curve, and S_v_ is the sample volume in µL. TAC values are converted and expressed in µmol per gram of fresh weight.

6 $$C=\left(\frac{Sa}{Sv}\right)=(\frac{\mu\, mol/\mu L}{g\, tissue/\mu L})$$

#### Chlorophyll content

Total chlorophyll content was determined on the basis of Winterman & de Mots^41^. Specifically, 100 mg of fresh whole aerial tissue (shoot) was taken from each treatment and immersed in 5 mL of ethanol at 96%. The samples were then triturated and left overnight to extract the pigments. The mixture was centrifuged for 3 min at 10,000 rpm. Absorbance at 654 was measured in a spectrophotometer (Jenway, 6715 UV). The estimation of total chlorophyll was obtained with Eq. (7) and expressed as milligrams of total chlorophyll per gram of fresh weight.

7 $$C=\left(\frac{1000 * A654\,nm}{39.8}\right)$$

#### Proline content

The proline content in tomato seedlings was assayed according to the standard method^42^. A standard curve was prepared with L-proline (≥ 99%). Furthermore, we prepared, diluted, and centrifuged whole aerial tissue samples to obtain 2 mL of filtrate, and we added 2 mL of acid ninhydrin and 2 mL of acetic acid glacial. A blank was prepared (same reagents without plant tissue). All samples were water bathed and boiled for 60 min, and immediately cooled with ice. Then, 4 mL of toluene was added and the solution was vigorously shaken. Finally, the absorbance in the chromophore was measured at 520 nm and the proline content was calculated using the Eq. (8) and expressed in µmol of proline per gram of fresh weight.

8 $$Proline \,content=\left(\frac{\frac{\mu g\, proline}{mL} *\, mL\, toluene}{115.5}\right)*\left( \frac{5}{g \,of \,sample}\right).$$

#### Nitrogen determination

Total nitrogen content was determined according to the persulfate digestion method^43^. In brief, a HI839800 reactor (HANNA instruments) and 10 mL of sulfuric acid were used to digest aerial and root tissues from tomato seedlings at 105 °C for 1–2 h. Persulfate powder pillows were added to p hydroxide digestion reagent vials with 0.5 mL of the digested samples (0.5 mL of deionized water for blank) were added. Then, vials were vigorously shaken for 30 s and placed in a heating reactor for 30 min at 105 °C and let to cool to room temperature. TN Reagent A was added, and vials were shaken for 30 s, while a 3-min reaction happened. After, TN Reagent B was added to the vials which were shaken for 15 s, while a 2-min reaction happened. Then, 2 mL were extracted and added to TN Reagent C vials. Program “394 N, Total HR TNT” was started in a DR 1900 spectrophotometer (Hach company). Finally, blank vial is inserted into the 16-mm cell holder and set as “zero”, then each sample nitrogen content is read in mg/L. The total nitrogen content estimation is converted to grams $(\frac{mg/L}{g\, tissue/L})$ and multiplied by 1000 to express in grams per kilogram of fresh weight.

### Metals determination

A screening of metals including macro and micronutrients in seeds and whole seedling tissues from tomato were determined. Prior to analysis, seeds and tissues were dried until constant weight and then subjected to acid digestion in a microwave oven system (Mars 6, CEM corporation). The digested samples were then diluted using deionized water and analyzed by inductively coupled plasma mass spectrometry (ICP.MS) in Agilent 7800 ICP-MS^44^. The elemental content is converted from ppb to milligrams per kilogram of dry weight.

### Statistical analysis

The germination quality, seedlings growth, biochemical parameters, and nitrogen content were statistically analyzed using one-way analysis of variance (ANOVA) with Origin Pro, considering that Se NPs concentration was the variable factor. Metal contents were also analyzed with Pearson correlation matrices. All pairwise multiple comparison procedures were conducted with Holm-Sidak method, and significant differences between treatments and control are based on a probability of *p* < 0.05, unless otherwise specified. Data are presented as mean ± standard errors (*n* = 4), unless otherwise specified.

## Results

### Characterization of Se NPs

**Figure 1:**
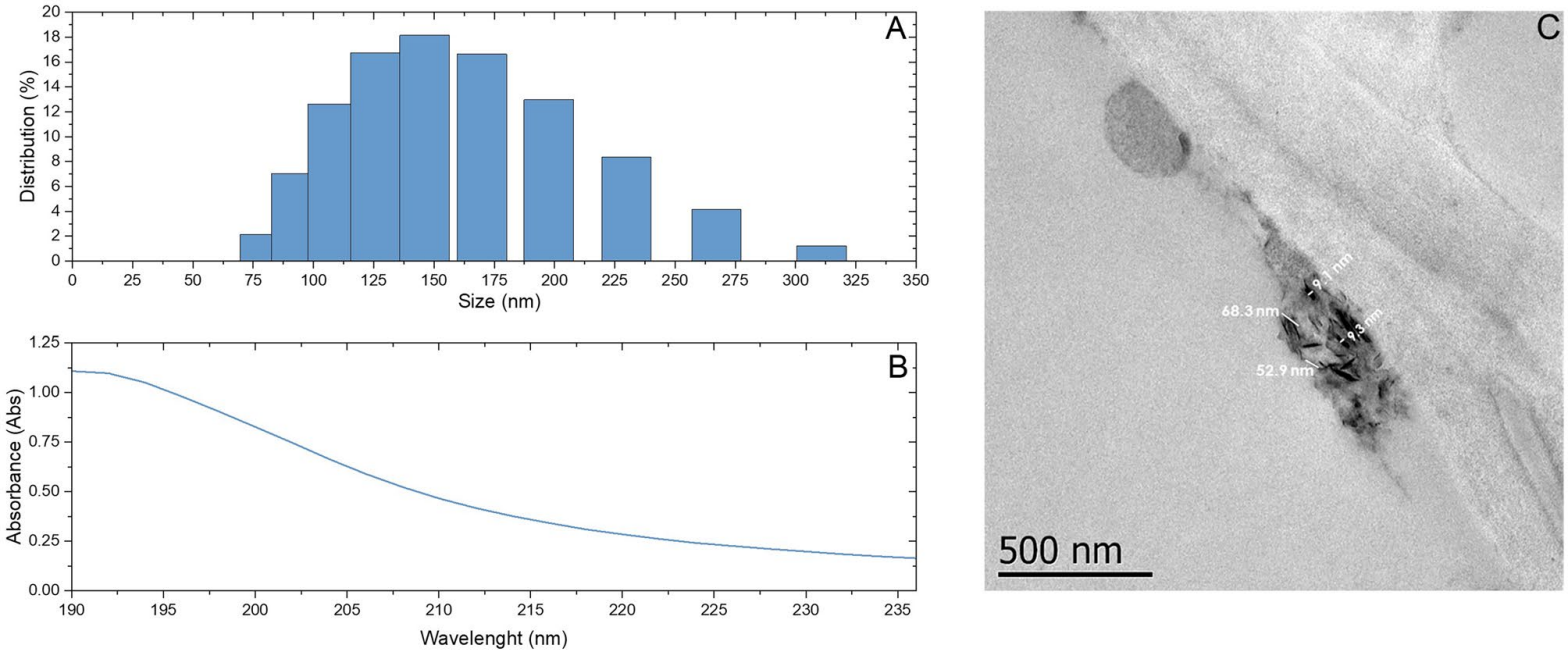
Characterization of Se NPs as function of size distribution, LSPR, and TEM micrographs. (**A**) DLS measurement indicated Se NPs size distribution from 75 to 320 nm (mean = 143.9 nm). (**B**) LSPR peak registered the highest absorbance in the UV-C region at 190 nm. (**C**) TEM micrograph of irregular shaped Se NPs (63.3 ± 8.1 nm length, 9.3 ± 1.5 nm width).

DLS measurements indicated that Se NPs mean size distribution was 143.9 nm (Fig. 1A) with a polydispersity index of 0.0908, which disagrees with TEM micrographs (63.3 ± 8.1 nm length, 9.3 ± 1.5 nm width). Differences in the size of Se NPs obtained by DLS and TEM can be by the possible agglomeration of the NPs in the solution measured through DLS. The LSPR peak (Fig. 1B), which is determined to understand the photocatalytic activity of the NPs, was detected in the UV-C region at 190 nm. TEM images revealed that Se NPs had an irregular shape (Fig. 1C), which can also contribute to differences in size of the agglomerates. The mean conductivity of Se NPs was 0.4077 mS/cm and the ζ potential was observed at -48.19 mV, indicating that Se NPs had a stable dispersion in water.

### Germination of tomato seeds primed with Se NPs

**Figure 2:**
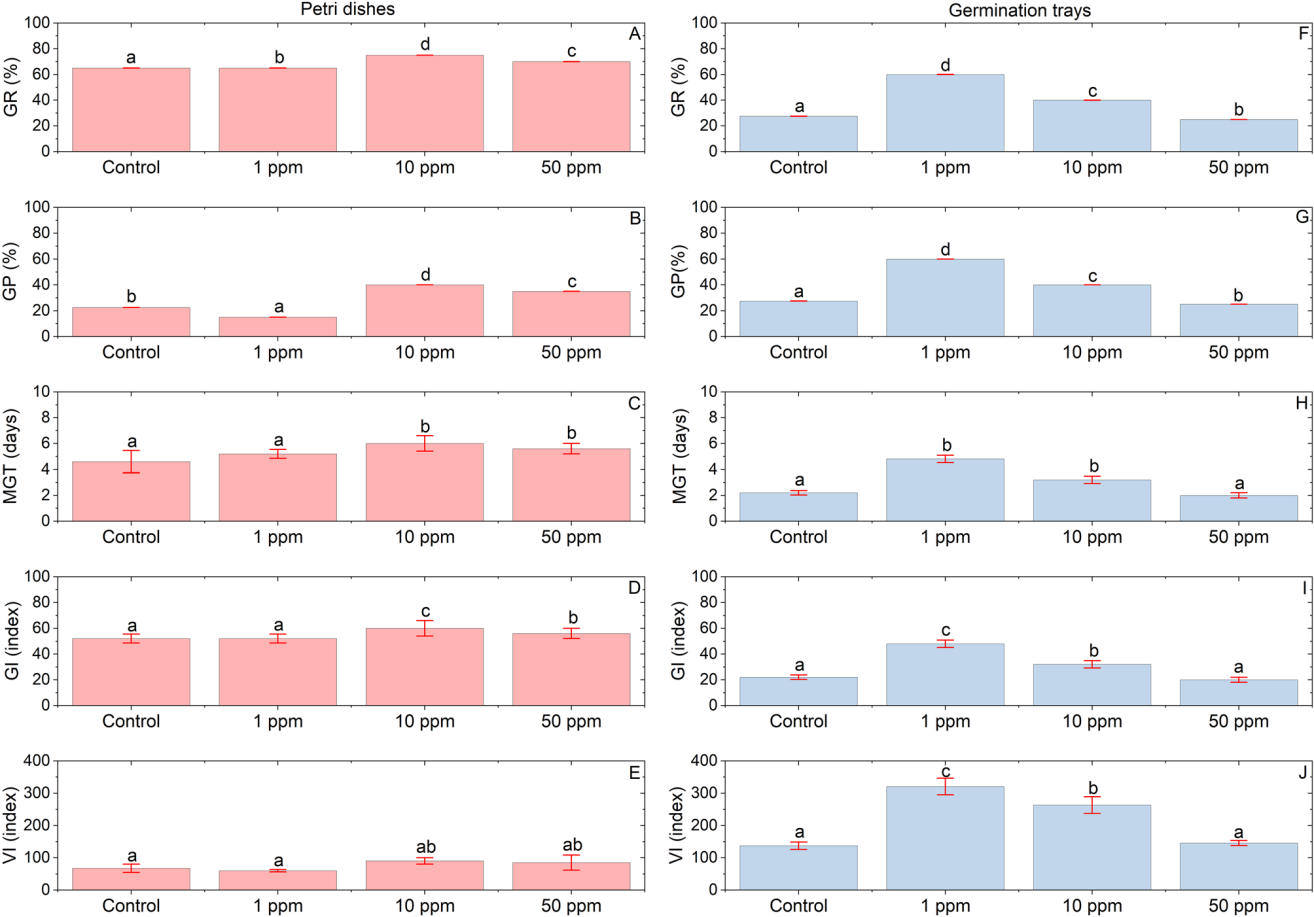
Germination quality of tomato seeds germinated in Petri dishes (left panels) and germination trays (right panels) as function of different Se NPs concentration. (**A**,**F**) Germination rate. (**B**,**G**) Germination potential. (**C**,**H**) Mean germination time. (**D**,**I**) Germination index. (**E**,**J**) Vigor index. Each result is a mean of 4 replicates. Statistical analysis was carried out using Holm-Sidak test separately for each germination method and parameter. Significant differences (*p* < 0.05) between means are represented by different letters in treatment bars. Red vertical lines with caps represent the ± standard error.

The data presented in Fig. 2 revealed that nanopriming tomato seeds with Se NPs improved the germination quality. In Petri dishes, nanopriming the seeds with 10 ppm increased the germination rate up to 10% as compared to control (Fig. 2A), while the germination potential increased up to 17.5%. Better results were obtained with trays with a lower dosage (Fig. 2B), where the germination rate (Fig. 2F) and potential increased up to 32.5% with 1 ppm (Fig. 2G), as compared to control. The mean germination time also increased accordingly for both methods due to the higher number of seeds germinating. This resulted in germination time of up to 6 days (Fig. 2C) in Petri dishes at 10 ppm, and 4.8 days in germination trays at 1 ppm (Fig. 2H). Furthermore, the germination index increased up to 13.33% in Petri dishes with 10 ppm (Fig. 2D), while trays presented a higher efficiency at 1 ppm, increasing the germination index up to 54.16% as compared to control (Fig. 2I). The vigor index was also significantly enhanced in trays (Fig. 2J), increasing up to 57.23% as compared to control. In Petri dishes, 10 ppm increased the vigor index up to 25.22% (Fig. 2E), however, the results were statistically similar to the control. In both germination methods and all parameters measured, the results obtained from nanopriming tomato seeds with 50 ppm resulted in either no statistical differences, or even decreases as compared to control groups.

### Fate of Se NPs within tomato primed seeds

**Figure 3:**
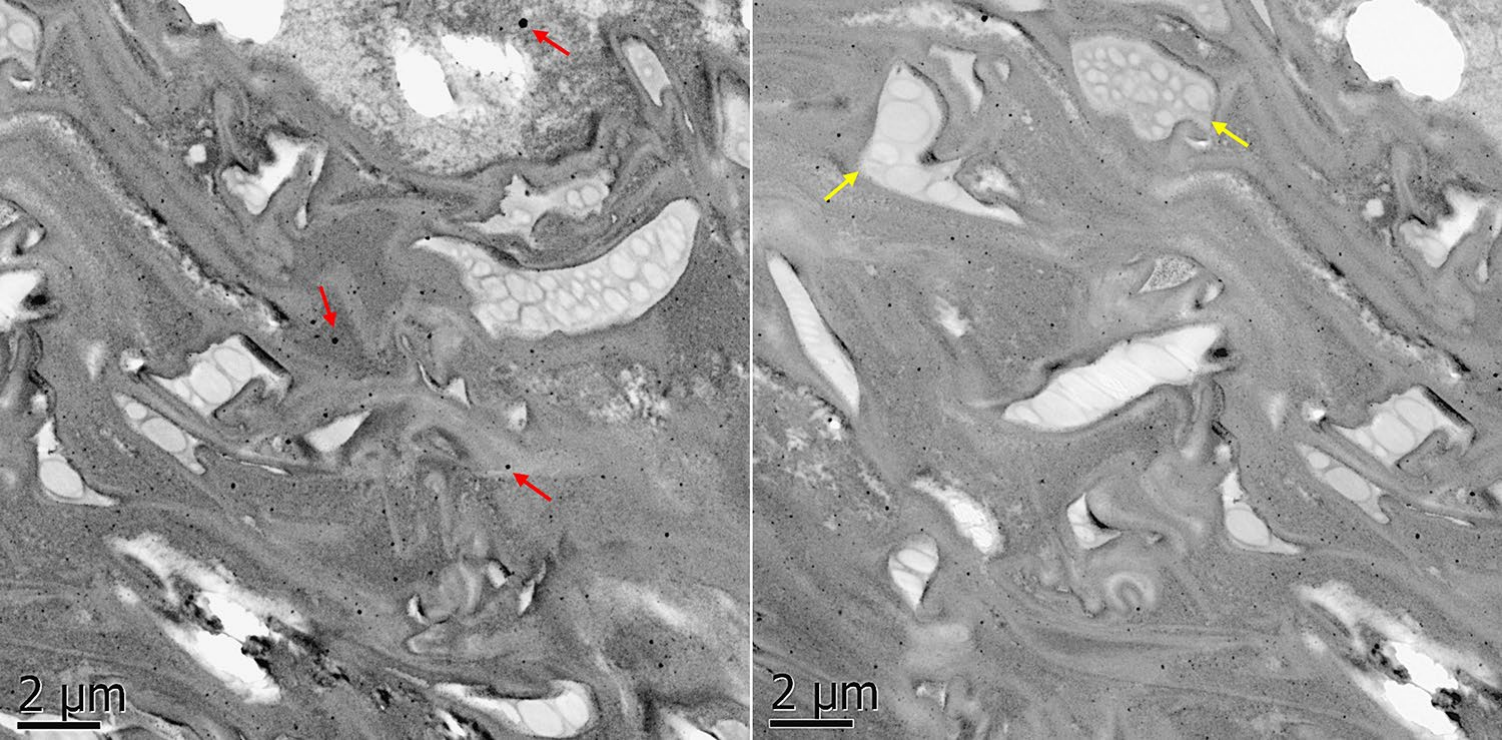
TEM micrographs confirmed the penetration of Se NPs through the seed coat of tomato nanoprimed seeds and their presence within the endosperm. Se NPs in the left micrograph are pointed by red arrow heads. Proplastids in the right micrograph are pointed by yellow arrow heads.

Figure 3 shows TEM micrographs tomato nanoprimed seeds exposed to Se NPs. The penetration of Se NPs through the seed coat was confirmed (Red arrows). Moreover, Se NPs can be observed close to the membrane of a proplastid within the seed, prompting unknown interactions and mechanisms in the biogenesis of chloroplasts and other organelles.

### Seedlings growth

**Figure 4:**
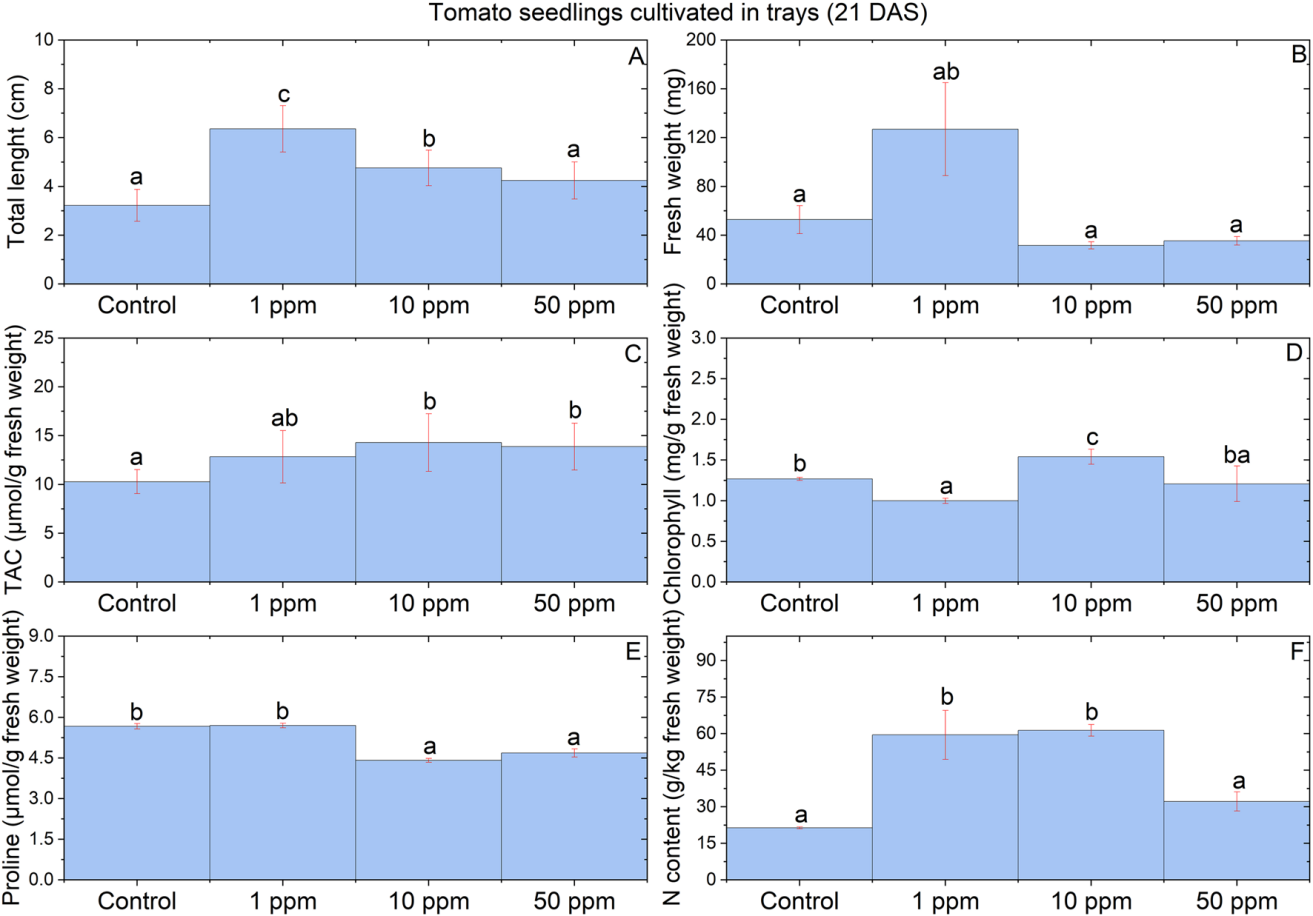
Physiological parameters of tomato seedlings cultivated in trays (21 DAS). (**A**) Length. (**B**) Fresh weight. (**C**) TAC. (**D**) Total chlorophyll content. (**E**) Proline content. (**F**) Total Nitrogen content. Each result is a mean of 4 replicates, statistical analysis was carried out using Holm-Sidak test separately for each parameter. Significant differences (*p* < 0.05) between means are represented by different letters in treatment bars. Vertical lines with caps represent the ± standard error.

The tomato nanoprimed seedlings germinated in trays presented significant increases in the total length (21 DAS). The total length increased up to 97.33% and 47.67% respectively for 1 and 10 ppm of Se NPs, as compared to control (Fig. 4A). However, NPs had no impact on the fresh weight of the seedlings (Fig. 4B).

#### Total antioxidant capacity

TAC values of whole aerial tissues in tomato nanoprimed seedlings (21 DAS) germinated in trays significantly increased when seed were exposed to10 and 50 ppm of Se NPs. As shown in Fig. 4C, results obtained with 1 ppm show statistical similarity to the treatments of 10 and 50 ppm, and control group. The highest TAC values were obtained for 10 ppm, with increases up to 38.97% as compared to control, while for 50 ppm the TAC of the tomato seedlings increased up to 34.84%.

#### Chlorophyll content

The total chlorophyll content obtained from whole aerial tissues in tomato nanoprimed seedlings (21 DAS) germinated in trays increased significantly but only with 10 ppm of Se NPs, as shown in Fig. 4D, the increases achieved up to 21.28%. Conversely, the treatment of 1 ppm decreased the total chlorophyll content up to 21.18%, while the treatment of 50 ppm showed similarities with both control and 1 ppm, but no significant reductions were noted.

#### Proline content

The proline content (an important stress marker) in whole aerial tissues of tomato nanoprimed seedlings (21 DAS) germinated in trays was significantly reduced when seed were exposed to 10 and 50 ppm of Se NPs. The most effective treatment was 10 ppm, with a decrease of 22.19%, while 50 ppm achieved a reduction of 17.40% as compared to control. Almost identical results were obtained from 1 ppm and control (Fig. 4E), showing no differences in the level of this osmolyte.

#### Nitrogen content

The nitrogen content in plants is associated with the growth of the seedlings and their photosynthetic activity. In tomato nanoprimed seedlings (21 DAS), significant increases were noted with 1 and 10 ppm of Se NPs. The best results were obtained with 10 ppm, where the nitrogen content increased up to 187.5%, while the increase obtained with 1 ppm was up to 178.9%, as compared to control. Statistically, there were no differences between control and 50 ppm, meaning that the N content remained at similar levels, as shown in Fig. 4F.

### Metal content

Se NPs induced significant variations of metals content for seeds and seedlings (21 DAS) (Table 1). In tomato nanoprimed seeds, the Se content increased accordingly to the treatments of Se NPs, 1 ppm (4.2%), 10 ppm (104.88%) and 50 ppm (657.48%). However, Ca and Cu significantly decreased their concentration in presence of Se NPs. The Ca content in seeds was reduced in all treatments between 31.95 and 40.23%, while the Cu content decreased between 16.75—32.5% (Table 1). Similarly, in tomato seedlings (21 DAS), Ca and Cu content decreased in all treatments in a range of 32.58–53.47%, and 39.34–58.17%, respectively. Furthermore, the Mo and K content also decreased between 42.86 and 57.14%, and 45.17–69.43%, respectively (Table 2). This tendency was not observed for Se content in the tomato seedlings, where all treatments resulted in either no significant differences with control. Furthermore, the antagonistic effects of Se with other mineral nutrients in tomato Se NPs primed seeds were confirmed by a Pearsons correlation matrix as shown in supplementary information (SI, Table S1) highlighting the antagonistic effects on K, Mn, and Cu. Conversely, Se NPs primed tomato seedlings (21 DAS) showed a synergistic correlation between the Se content and important nutrients, such as Mg, K, Fe and Zn (SI, Table S2).

**Table 1:** Effect of Se NPs on Se and other mineral nutrient content in tomato primed seeds (mg/kg dry weight) Data represent the mean and standard error of three experimental replicates.

	Control	1 ppm	10 ppm	50 ppm
Se	0.25 ± 0.06	0.26 ± 0.90	0.50 ± 0.80	1.86 ± 0.06*
Na	2306.75 ± 182.10	2528.10 ± 100.20	2651.76 ± 30.20	2442.70 ± 137.60
Mg	4422.16 ± 296.40	4178.74 ± 27.30	4292.91 ± 112.60	3842.43 ± 245.80
Al	88.80 ± 6.40	110.23 ± 23.80	75.06 ± 10.20	82.22 ± 4.40
K	4817.94 ± 240.60	5241.04 ± 105.03	5058.45 ± 145.80	3787.62 ± 457.10
Ca	1458.49 ± 97.40	992.42 ± 40.20*	871.65 ± 43.04*	890.13 ± 53.70*
Ti	6.08 ± 1.05	7.77 ± 2.39	5.25 ± 0.76	5.53 ± 0.21
Mn	73.20 ± 5.50	62.49 ± 1.25	62.99 ± 2.90	54.02 ± 4.40
Fe	217.61 ± 11.70	218.50 ± 7.70	201.06 ± 7.30	184.24 ± 15.70
Cu	21.55 ± 0.90	17.92 ± 0.35	17.99 ± 0.60*	14.52 ± 1.20*
Zn	141.48 ± 4.60	183.99 ± 32.90	139.21 ± 6.70	116.44 ± 15.60
Mo	0.87 ± 0.07	0.75 ± 0.02	0.69 ± 0.02	0.64 ± 0.04

Statistical significance is indicated by * (p < 0.05) (Holm-Sidak versus control).

**Table 2:** Effect of Se NPs on Se and other mineral nutrient content in tomato primed seedlings (mg/kg dry weight) Data represent the mean and standard error of three experimental replicates.

	Control	1 ppm	10 ppm	50 ppm
Se	0.20 ± 0.06	0.04 ± 0.02	0.29 ± 0.01	0.20 ± 0.10
Na	1774.27 ± 160.60	740.19 ± 80.90*	1723.87 ± 101.20	1950.34 ± 222.03
Mg	3439.24 ± 354.70	1154.47 ± 126.70	1307.65 ± 245.60	2080.93 ± 956.10
Al	1147.60 ± 222.06	274.33 ± 71.10	258.17 ± 112.90	570.71 ± 431.80
K	2265.91 ± 300.01	692.62 ± 35.40*	766.05 ± 56.70*	1242.46 ± 292.40*
Ca	2200.66 ± 102.80*	1158.98 ± 77.01*	1023.97 ± 51.90*	1483.55 ± 286.40*
Ti	122.73 ± 22.70	24.88 ± 6.09	24.24 ± 10.20	51.16 ± 39.20
Mn	23.20 ± 1.60	13.07 ± 1.80*	9.38 ± 0.90*	12.28 ± 4.10
Fe	1117.24 ± 218.40	231.29 ± 52.01	232.21 ± 88.30	498.66 ± 358.40
Cu	10.05 ± 0.60	4.23 ± 0.20*	6.06 ± 0.20*	6.09 ± 0.70*
Zn	24.17 ± 1.50	12.99 ± 1.90	17.34 ± 0.80	50.27 ± 27.20
Mo	0.14 ± 0.01	0.06 ± 0.01*	0.08 ± 0.01*	0.07 ± 0.01*

Statistical significance is indicated by * (p < 0.05) (Holm-Sidak versus control).

## Discussion

Nanopriming tomato seeds with Se NPs resulted in significant increases in the germination quality in trays and Petri dishes, enhancing the germination rate, vigor, and germination indexes. In trays, the effectiveness of Se NPs as nanopriming agents exceeded the results obtained in Petri dishes, increasing the germination rate by 32.5% with 1 ppm and the germination and vigor indexes between 54.16 and 57.23% for all Se NPs concentrations tested. Furthermore, the positive results obtained in trays were achieved with 1 ppm of Se NPs, whereas the positive effects of Se NPs in Petri dishes were registered with 10 ppm. During the appearance of the radicle, the hypocotyl and epicotyl of seeds germinated in Petri dishes were deformed due to the absence of substrate, resulting in death of the seedlings during transplant. These results and the higher efficiency achieved by trays prompted the further experiments to be conducted on seedlings germinated in trays.

The resulting germination quality for each method can be due to small differences in environmental factors, such as temperature, moisture, light conditions, and the substrate^45^. The results obtained are in accordance with recent nanopriming studies with Se NPs. For example, germination and vigor of tomato nanoprimed seeds affected by late blight disease increased by 22 and 59.86% with 100 ppm of Se NPs, respectively^31^. Moreover, in heat-stressed tomato plants, the concentrations of Se NPs with the best results on growth parameters were found to be 5 and 10 ppm, while 20 ppm showed signs of phytotoxicity^30^. In Barley (*Hordeum vulgare*) nanoprimed seeds with Se NPs, the best treatment for the germination quality in Petri dishes was also 5 ppm, while 10 ppm resulted in the highest number and thickness of roots^46^. In accordance with these studies, we found similar concentrations of Se NPs to have the best results in our nanopriming experiment for tomato seeds. The main difference with reported studies is that we did it without induced stress conditions. Under non-stressed conditions, the best treatments to increase the germination quality in nanoprimed tomato seeds were 1 ppm and 10 ppm in trays and Petri dishes, respectively. Whereas in trays, the treatment of 10 ppm achieved the best results on tomato seedling growth.

Improving the germination quality can result in a more efficient nutrient and water uptake of seedlings, enhancing the resilience to biotic and abiotic stress^12^. Even though Se is a nonessential element for plants^47^, previous studies have reported that Se NPs can help to improve the germination, growth, and stress resilience of different crops^38,48,49^. Although the exact mechanisms of such improvements with Se NPs are still unknown, it has been proposed that the germination rate of different seeds can increase due to the NPs creating nanopores through their penetration, which consequently increases the imbibition of water by the seeds. Also, the selective permeability of the seed surface pores can help to internalize or restrict the uptake of NPs^50^. This could explain why we observed Se NPs within the endosperm of tomato nanoprimed seeds. Furthermore, a positive linear correlation was found between the treatments (1, 10, 50 ppm of Se NPs) and the seed Se content (SI, Fig. S1), while TEM micrographs revealed Se NPs near a proplastid. Furthermore, transport of Se NPs within plant tissues through the symplastic pathway is possible, due to their possible penetration through the plasma membrane^51^. This means that Se NPs not only penetrate the seed coat but also the embryo, prompting unknown interactions with proplastids, which can transform into chloroplasts and other organelles during biosynthesis. In this last bioprocess, the production of chlorophyl, antioxidants (including antioxidant enzymes such as superoxide dismutase, catalase, glutathione peroxidase, and antioxidants such as glutathione, ascorbic acid, 4-aminobutyric acid, α-tocopherol, ferulic acid, amongst others)^52^, proline, and other important compounds can occur. Such metabolic activities resulted in a higher total chlorophyll content, and TAC in aerial tissues of nanoprimed tomato seedlings, while the proline content (an important osmolyte) was significantly reduced in nanoprimed tomato seedlings.

There was positive correlation of total chlorophyll and TAC with the seed Se content (SI, Fig. S4), as well as a negative correlation of proline content with seed Se content (SI, Fig. S4). These correlations are important because chlorophyll is an essential molecule associated with photosynthetic activity and growth of the seedlings^53^. Besides, the antioxidative system in plants is a complex multilevel network, including enzymes of the glutathione-ascorbate cycle which serve to maintain homeostasis within the cell and counteract reactive oxygen species (ROS), among others^54^. Proline accumulation also plays important roles in stress tolerance for many plants species^55^, including the roles in vegetative growth of seedlings as a metabolite and signal molecule^56^. Although the exact mechanisms and interactions in which Se NPs can increase the total chlorophyll and TAC content, and reduce the proline content remain unknown, we speculate that the results obtained might be explained by Se NPs increasing the content of glutathione in tomato. More than 90% of total glutathione content is in reduced form (GSH), reduced by the NADPH-dependent activity of glutathione reductase (GR), while less than 10% is in its oxidized form which is glutathione disulfide (GSSG)^57^. The Se-dependent glutathione peroxidase (GPX) is involved in the plant metabolism, redox signaling, and defense systems^58^. In the ascorbate–glutathione cycle, GSH is oxidized to GSSG, and then reduced again to GSH by GR. This cycle involved both the reduced and oxidized forms of glutathione, and the balance between GSH and the ascorbate content maintains the cellular redox state in plants^59^.

Bringing together three monitored parameters such as TAC, chlorophyll, and proline, we found that all three and Se are related with glutamate which is essential for the glutathione cycle. In fact, it has been reported that ionic Se can increase the content of glutathione in tomato leaf tissues^60^. Glutathione is one-third glutamate, which is vital in the metabolic pathway of chlorophyll biosynthesis in the chloroplasts and thylakoid membranes^53^. Glutathione is a non-enzymatic antioxidant that prevents lipid peroxidation and protects the plasma membrane^61^. Furthermore, chloroplasts require glutathione reductase to maintain an efficient photosynthesis and plant growth^62^. Therefore, Se NPs might increase the total chlorophyll content by increasing metabolites involved in the metabolic pathway of chlorophyll biosynthesis. In fact, it has also been proposed that Se can follow the glutathione sulfhydryl transferase (GST) transfer pathway, due to the evidence that Se and anthocyanins shared the same transcription factors in wheat^63^. In addition, glutathione transferases (GSTs) serve as non-catalytic carrier proteins involved in the vacuolar uptake of anthocyanins in tomato and other crops^64^. Furthermore, it was found that individual anthocyanins have important contributions to TAC in other crops, with pelargonidin 3-glucoside (Pg-3-G) displaying the highest TAC values^65^. The enzymes of the glutathione-ascorbate cycle are the most closely related to the antioxidant system in plants^54^. This could explain the significant increases in TAC by nanopriming tomato seeds with Se NPs and can be closely related to the total chlorophyll content increases. Furthermore, the glutamate pathway accounts for the most proline accumulation in plants during osmotic stress^66^. The chloroplasts, mitochondria and cytoplasm are involved in the proline metabolism^67^. As well as in the chlorophyll and antioxidants metabolisms^53,65^. Meaning that interactions of Se NPs within the cell could result in a reduction in proline content, which is in accordance with the increases in total chlorophyll content and TAC. This could be further supported by the evidence of antagonisms of Se NPs with other important mineral nutrients. The TAC values and proline content were obtained from whole aerial tissues because the biomass of the roots was minimum, however, future studies should consider the roots values and whole seedling values to properly assess the effects of Se NPs on the seedling development.

Through Pearson correlation coefficient matrices, we observed that the Se content resulting from nanopriming tomato seeds with Se NPs resulted in antagonistic effects over essential macro and microelements in the seed, such as Mg, K, Mn, Fe, Cu and Mo. This is consistent with other studies that have reported antagonistic effects of ionic Se over Mo, Fe, Mn, and Cu content in tomato roots^60^. Furthermore, the flow direction of Se within plant tissues coincides with the transport pathways of S^68,69^, while antagonisms of Se with S, and As, have been reported early in the previous century^70^. Also, angiosperms such as tomato, tend to have similar shoot Se/S quotients, while Se and S share the same primary metabolism^71^. All of this might be explained by the chemical similarities of Se with S and As^72^. Although the consequences of such antagonistic effects of Se with other important nutrients are unclear, it is evident that the increases in germination quality are not related to the interaction with other nutrients. Conversely, synergistic effects of Se content in nanoprimed tomato seedlings were found with important nutrients, such as Mg, K, Ca, Fe, and Zn. The only element in which antagonistic effects of Se persisted was Mo. Furthermore, the Se content in the seedlings differed significantly with the Se content in the seeds, we speculate this was caused by the exudation mechanisms of the tomato seedlings expelling Se NPs into the substrate, as well as the seedlings not internalizing the total amount of Se that penetrated the endosperm^73^.

Although it remains unclear which are the exact mechanisms of Se NPs promoting the germination and growth of tomato seedlings, metabolomics and other “omics” have been proposed to elucidate the different expressions of metabolites in plants, which could explain the positive effects of NPs in the phenotype of such plants^74^. Metabolomics can help to understand the differences in metabolic pathways of carbohydrates, vitamins, amino acids, glucosinolates, amongst others, which can be helpful to assess the physiological status of the plants^75^. While this work focused on germination and early vegetative growth of tomato, longer life cycle studies which implement genomics, transcriptomic, proteomics, and metabolomics, are recommended for future work, to completely understand the response of tomato plants to Se NPs, and their mechanisms and interactions which prompt such responses.

## Conclusions

In this work, nanopriming tomato seeds with Se NPs effectively increased the germination quality in both germination methods trays and Petri dishes. The treatments of 1 and 10 ppm of Se NPs exhibited the best performance on germination for trays and Petri dishes, respectively. Trays showed a greater germination quality than Petri dishes and were selected for further experimentation. Nanoprimed tomato seedlings showed the best growth with 10 ppm of Se NPs, significantly increasing their total length. The same treatment resulted in the best results for total chlorophyll content and antioxidant capacity in whole aerial tissues. Furthermore, such increases in vegetative growth are consistent with the significant reduction in proline content. Various studies have reported a reduction of proline content, as well as increases in total chlorophyll content in different crops with NPs of different nature, but information of the impact of seed priming with Se NPs in tomato without induced stress conditions is scarce. We conclude that Se NPs can be used as an effective nanopriming agent to increase the germination quality and growth of tomato, because of the positive impact previously described on the germination with 1 ppm and the seedlings growth with 10 ppm. However, the mechanisms in which Se NPs prompt such increases in the germination quality and seedling growth are unknown, therefore, it is essential to elucidate the intricacies of Se NPs interactions with the different plant systems before properly implementing the use of Se NPs in sustainable agriculture. To achieve this, quantitative studies with metabolomics addressing the glutathione pathway of tomato plants treated with Se NPs are needed. Furthermore, genomics, transcriptomics and proteomics can also be utilized to understand the interactions between Se NPs and plant tissues on a deeper scale, while full life cycle studies of crops can also be useful to determine if the Se NPs can impact chloroplasts and other organelles within plant tissue as function of time.

### Supplementary Information

Supplementary Information.

## Data Availability

All data generated or analyzed are included within this article or in supplementary information. The data are available from the corresponding author upon reasonable request.
