# Supplementary material for: Impact of seed priming with Selenium nanoparticles on germination and seedlings growth of tomato
Source: Sci Rep. 2024 Mar 20;14:6726. doi: 10.1038/s41598-024-57049-3 (PMC10954673; doi:10.1038/s41598-024-57049-3)
Supplement: Supplementary file 1 — Supplementary Information. [file 41598_2024_57049_MOESM1_ESM.pdf]

Impact of seed priming with Selenium nanoparticles on germination and seedlings growth of tomato

Supplementary Information

Ezequiel García-Locascio[1] , Edgardo I. Valenzuela[1] , and Pabel Cervantes-Avilés [1,*]

[1] Tecnologico de Monterrey, Escuela de Ingeniería y Ciencias, Reserva Territorial

Atlixcáyotl, Puebla, Pue, México CP 72453

* **Correspondence:** pabel.cervantes@tec.mx; Tel.: +52 222 303 2000

Table of contents

Section name	Page
1. Table S1. Pearson's correlation coefficient matrix of Se NPs in tomato nanoprimed seeds	2
2. Table S2. Pearson's correlation coefficient matrix of Se NPs in tomato nanoprimed seedlings	3
3. Fig. S1. Relation between treatments and Se content in the seed	3
4. Fig. S2. Relation between germination parameters in seeds germinated in Petri dishes and Se content in the seed.	4
5. Fig. S3. Relation between germination parameters in seeds germinated in trays and Se content in the seed.	5
6. Fig. S4. Relation between total chlorophyll, Total Antioxidant Capacity, and proline with Seed Se content in tomato seedlings germinated in trays.	6

Table S1. Pearson's correlation coefficient matrix of Se NPs in tomato seeds

	Se	*Na*	*Mg*	*Al*	*K*	*Ca*	*Ti*	*Mn*	*Fe*	*Cu*	*Zn*	*Mo*
Se	1											
Na	-0.72	1										
Mg	-0.92	0.93	1									
Al	-0.46	-0.29	0.08	1								
K	-0.99	0.74	0.94	0.43	1							
Ca	-0.50	-0.25	0.13	0.99	0.48	1						
Ti	-0.54	-0.21	0.17	0.99	0.51	0.99	1					
Mn	-0.98	0.84	0.98	0.28	0.99	0.33	0.36	1				
Fe	-0.92	0.40	0.71	0.76	0.91	0.79	0.82	0.83	1			
Cu	-0.99	0.82	0.97	0.31	0.99	0.35	0.39	0.99	0.85	1		
Zn	-0.84	0.23	0.58	0.86	0.83	0.89	0.90	0.73	0.98	0.75	1	
Mo	-0.93	0.41	0.72	0.75	0.92	0.78	0.81	0.84	0.99	0.86	0.98	1

Table S2. Pearson's correlation coefficient matrix of Se NPs in tomato seedlings

	Se	*Na*	*Mg*	*Al*	*K*	*Ca*	*Ti*	*Mn*	*Fe*	*Cu*	*Zn*	*Mo*
Se	1											
Na	0.59	1										
Mg	0.98	0.75	1									
Al	0.99	0.61	0.98	1								
K	0.98	0.73	0.99	0.99	1							
Ca	0.97	0.40	0.90	0.97	0.92	1						
Ti	0.99	0.63	0.98	0.99	0.99	0.96	1					
Mn	0.37	-0.52	0.16	0.36	0.19	0.57	0.33	1				
Fe	0.99	0.65	0.99	0.99	0.99	0.96	0.99	0.31	1			
Cu	0.46	0.99	0.64	0.47	0.61	0.25	0.50	-0.65	0.52	1		
Zn	0.99	0.72	0.99	0.99	0.99	0.92	0.99	0.21	0.99	0.60	1	
Mo	-0.19	0.67	0.02	-0.18	-0.01	-0.41	-0.15	-0.98	-0.13	0.78	-0.02	1

1. Relation between treatment and Se content in the seed

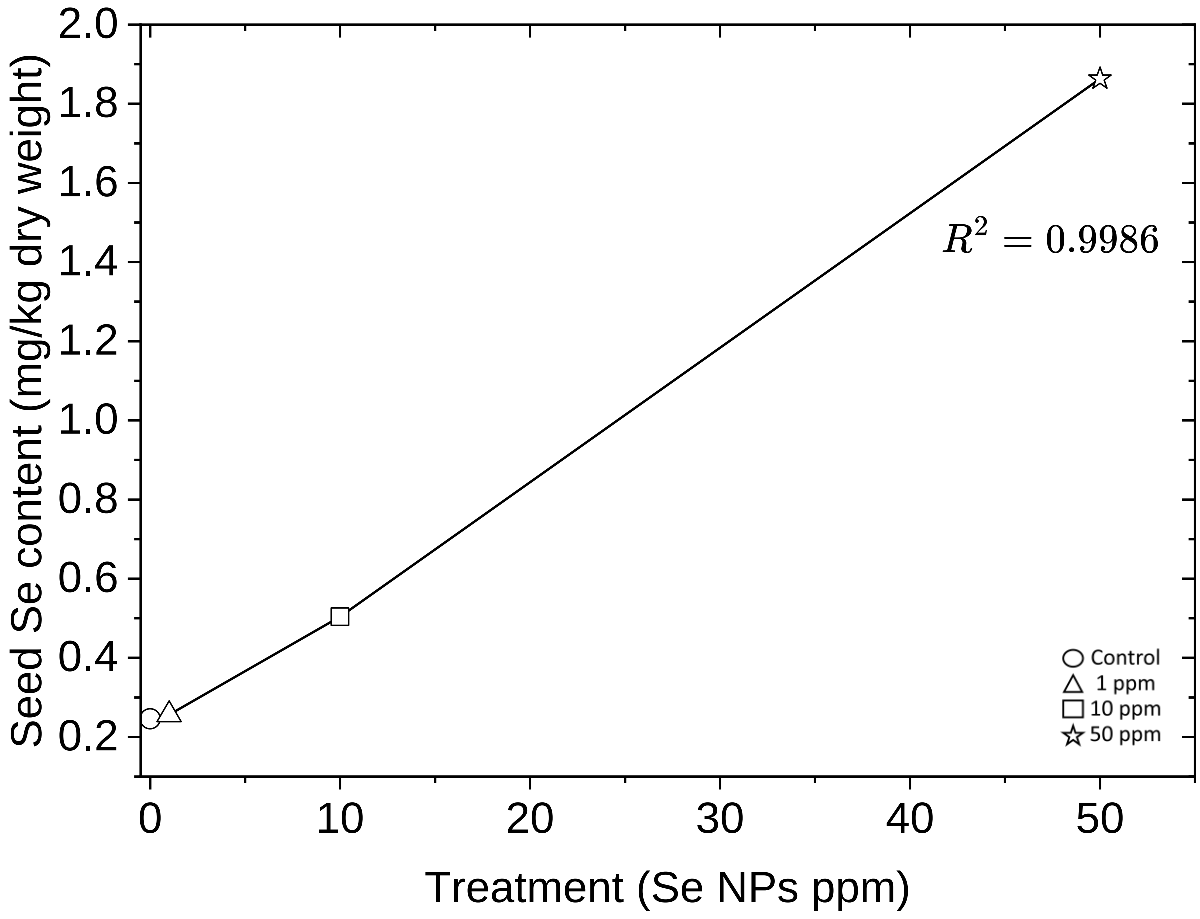

Figure S1. Linear correlation of Seed Se content (mg/kg dry weight) with treatments of Se NPs (1, 10 and 50 ppm) in tomato seeds.

2. Relation between germination parameters in seeds germinated in Petri dishes (germination rate, germination potential, mean germination time, germination index & vigor index) and Se content in the seed.

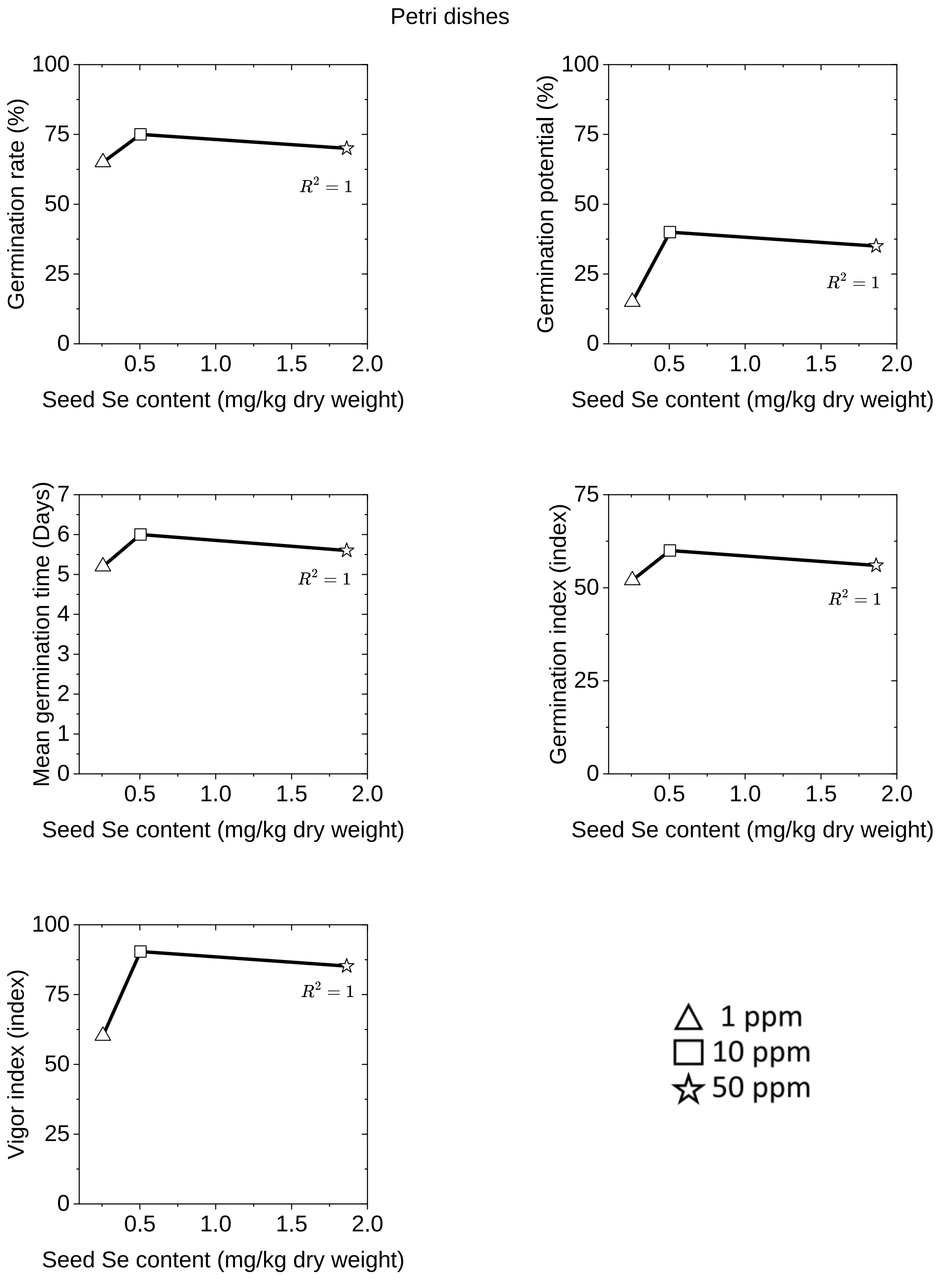

Figure S2. Polynomial correlation of Seed Se content (mg/kg dry weight) with parameters of germination quality in tomato nano-primed seeds germinated in Petri dishes.

3. Relation between germination parameters in seeds germinated in trays (germination rate, germination potential, mean germination time, germination index & vigor index) and Se content in the seed.

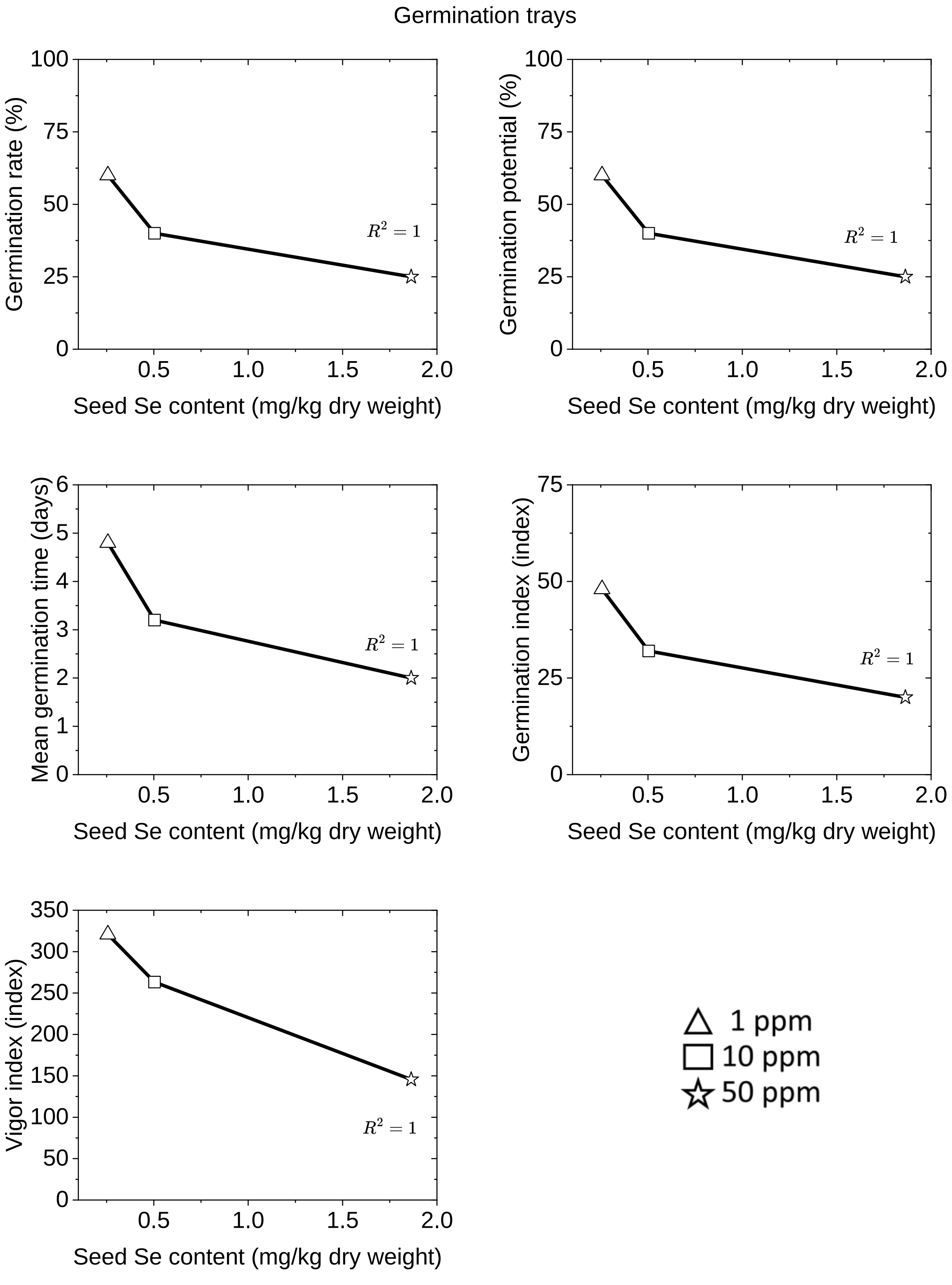

Figure S3. Polynomial correlation of Seed Se content (mg/kg dry weight) with parameters of germination quality in tomato nano-primed seeds germinated in Trays.

4. Relation between total chlorophyll, Total Antioxidant Capacity, and proline with Seed Se content in tomato seedlings germinated in trays.

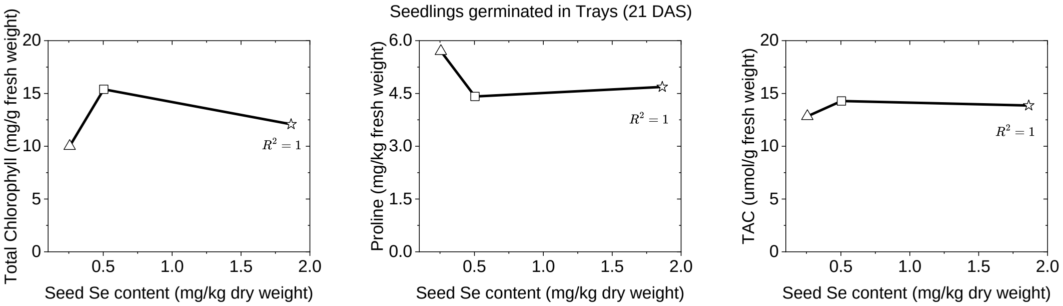

Figure S4. Polynomial correlation of Seed Se content (mg/kg dry weight) with total antioxidant capacity, total chlorophyll content, and proline content.
